# Total-Body PET: Will It Change Science and Practice?

**DOI:** 10.2967/jnumed.121.263481

**Published:** 2022-05

**Authors:** Austin R. Pantel, David A. Mankoff, Joel S. Karp

**Affiliations:** Department of Radiology, University of Pennsylvania, Philadelphia, Pennsylvania

Total-body (TB) PET scanners with an axial field of view (AFOV) of at least 60 cm can image dynamic radiopharmaceutical distributions in the whole body. Although the benefits of the fine temporal sampling and dramatic increase in system sensitivity afforded by TB PET imaging have been postulated for some time, it was not until the last few years that such systems have become available in both clinical and research settings. This article summarizes what we have learned in this short time and explores the long-term impact of TB PET.

## INSTRUMENTATION

### Existing Devices

Two human TB PET scanners became operational in 2018: United Imaging’s uEXPLORER scanner (AFOV, 194 cm), initially installed at Shenzhen Hospital in China and at the University of California Davis (*1*), and the PennPET Explorer scanner (AFOV, 64–142 cm), built and installed at the University of Pennsylvania (*2*). In 2020, Siemens introduced the Biograph Quadra scanner (AFOV, 106 cm), installed at Bern Hospital in Switzerland (*3*). Compared with PET scanners with a standard AFOV (16–30 cm), the total sensitivity grows significantly as the axial length increases, up to 50 times for a 2-m axial length, whereas the peak sensitivity gain for a point (or organ) reaches a maximum of about 2.5–3 times at 80–120 cm (depending on the patient’s body mass index) (*4*); longer systems maintain this peak sensitivity over a wider axial range. Although the underlying technology for TB PET scanners was already available, there were many challenges to make these systems robust, to efficiently handle the very large datasets, and to optimize the data acquisition and processing of oblique coincidence pairs. As is evident from the many hundreds of TB PET studies performed to date, the data correction and image reconstruction methods result in high-quality, quantitative images with improved accuracy of kinetic parameter estimation (*5*). The imaging protocols have been adjusted to leverage the enhanced performance, with no major problems or significant failures discovered in these first-generation TB PET scanners.

### Future Directions

The key challenge to wider dissemination of TB PET systems is cost, which is determined largely by the AFOV and the volume of the detectors. One can argue that the increased cost is justified, leading to benefits in research knowledge and clinical diagnostics, potentially with increased patient throughput. Or, we can consider ways to reduce the cost. Notably, the PennPET Explorer was designed to be scalable in axial length to allow for a choice in matching the benefits of TB PET for particular applications. In addition, given a preferred AFOV, the number of detectors used in the design of the system may be reduced, taking advantage of the huge redundancy of data used for 3-dimensional image reconstruction. This idea has been explored in several simulation studies (*6* *,* *7*) and has been demonstrated in practice with the PennPET Explorer, which has operated with inter-ring gaps that correspond to 30% of the detector’s active length (*2* *,* *7*). Alternatively, the cost of the detector can be significantly reduced by using a less expensive material (*8*) than the lutetium-based scintillators used in all modern PET/CT scanners. Efforts are also under way to develop systems with improved performance through the use of monolithic detectors to provide improved spatial resolution throughout the imaging field of view (*9*).

## CLINICAL IMAGING WITH TB PET

Clinical translation of TB PET has long been a goal in the development of these scanners. In only the short time since implementation, many of the proposed clinical applications have been borne out.

### Improved Detection of Disease

The gain in sensitivity and resultant improved image quality with TB PET can be leveraged to improve the ability to stage or restage disease and increase diagnostic confidence. For example, the PennPET Explorer detected a paracardiac ^18^F-FDG–avid lymph node that was seemingly occult on the standard-of-care PET (*2*). Because TB PET scanners provide significantly better image quality, the increased lesion contrast and detection may necessitate new interpretation paradigms to integrate into preexisting imaging criteria (e.g., the Deauville criteria) while not losing specificity (e.g., overcalling benign lymph nodes that can be more easily visualized).

### Optimization of Scan Protocols

The increased sensitivity of TB PET can be leveraged to achieve images of diagnostic quality with less injected activity or shorter scans. Images obtained with 25 MBq (0.7 mCi) of ^18^F-FDG were obtained on the uEXPLORER (*1*), and ^18^F-FDG PET scans (clinical dose) with a 2-min duration proved satisfactory on the prototype PennPET Explorer (*2*). Shorter scans can increase clinical throughput and minimize the effects of patient motion—both gross movement and internal organ motion. Using a low injected activity enables imaging of tracers that are difficult to produce (^68^Ga-DOTATATE), serial imaging of patients with a low injected activity, or imaging of children. In human translation of novel tracers, TB PET imaging could inform the scanning protocol. These instruments may have particular use for tracers with relatively fast kinetics (e.g., simultaneous early imaging of the abdomen and pelvis to optimize lesion contrast with ^18^F-fluciclovine before washout) or slow kinetics (e.g., delayed imaging to leverage increased trapping of ^18^F-FDG in malignancy or improved lesion contrast with prostate-specific membrane antigen tracers in prostate cancer). Indeed, on the uEXPLORER at the University of California Davis, a 2-h uptake time is routinely used for ^18^F-FDG PET.

### Nononcologic Applications

For clinical applications that do not require full-body coverage, such as dedicated cardiac or brain imaging, the single-organ sensitivity gains of TB PET scanners without axial coverage of the entire body may prove particularly valuable while mitigating cost. Superb image quality has been demonstrated with all new systems, noting again that increasing the AFOV beyond approximately 80–100 cm does not benefit single-organ imaging, providing an opportunity to match the AFOV with the intended application. Moreover, for high-sensitivity imaging of relatively static processes, such as leukocyte imaging of infection at 24 h or later with a ^89^Zr-labeled minibody, TB PET scanners without full-body coverage may still be used to image the total body at multiple bed positions.

## RESEARCH INVESTIGATIONS WITH TB PET

The technical advantages afforded by TB PET enable unique research applications of PET that are difficult, if not impossible, with standard-AFOV systems. A range of applications is described here.

### Better Definition of Radiopharmaceutical Kinetics and Biodistribution

TB PET provides a unique tool for characterizing the behavior of new imaging agents or understanding the nuances of existing agents (*10*). For first-in-humans studies of novel radiopharmaceuticals, TB PET may be leveraged to study pharmacokinetics in both diseased and normal tissues, to estimate radiation dosimetry, and to guide optimal acquisition protocols. The ability to image the biodistribution of the radiotracer late after injection can provide a powerful tool to estimate the delivered radiation dose for diagnostic–therapeutic theranostic pairs. TB PET may also provide unique insights into commonly used radiotracers. For example, kinetic analysis of dynamic ^18^F-FDG images revealed nuanced differences in regional kinetics in both normal tissues and tumors. On the PennPET Explorer, markedly delayed imaging of ^18^F-FDG out to 10 half-lives clearly demonstrated the late loss of tracer in the normal brain due to dephosphorylation of FDG-6P (*2*). This ability could have clinical utility in distinguishing brain tumors (which often do not dephosphorylate FDG-6P) from normal brain tissue.

### Novel Analysis of Dynamic PET Data

The acquisition of large volumes of 4-dimensional data (3-dimensional plus time) lends itself to, and even necessitates, innovative approaches to image analysis. The inclusion of both diseased and normal tissue, and differences in radiopharmaceutical transport and metabolism between different tissues, may require adjusting the kinetic model to different tissues (*11*) or considering dynamic PET data as a spatially varying mixture of characteristic kinetic curves. Model-free approaches to estimating key components such as tracer delivery and retention may offer computational efficiency and linear scaling, which can be especially helpful for large 4-dimensional datasets. Early efforts using artificial intelligence or data science on 4-dimensional data to predict survival by characterizing tissue heterogeneity have shown promise (*12*). Finally, image reconstruction algorithms that consider pixel time course as part of the image reconstruction process may offer improved image quality and quantitative accuracy (*13*).

### Applications to Systems Biology

**FIGURE 1.**
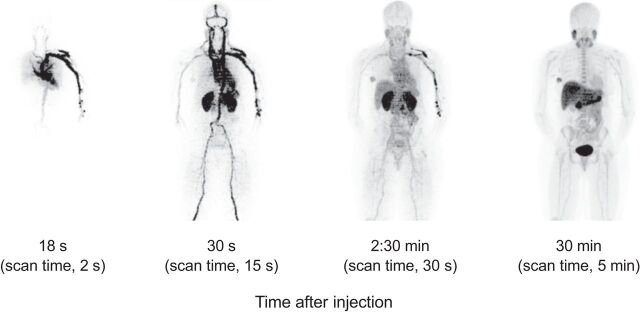
Illustration of TB PET imaging, showing dynamic sequence at representative postinjection times and scan times. Breast cancer patient was imaged with ^18^F-fluoroglutamine on PennPET Explorer to quantify glutamine metabolism.

Perhaps most exciting is the ability of TB PET to characterize human systems biology and to look at multisystemic interactions. Such opportunities include studying the heart–brain axis, the role of liver metabolism in drug addiction, and the interaction between tumors and host tissues such as immune reactions. A preclinical study of the interaction of bone metabolism with other organ systems provides a specific example of a study possible with TB PET but not with standard scanners (*14*). The ability to combine multiple PET tracers in a single study can be improved by TB PET imaging and offers the possibility of studying time-varying processes that require closely timed PET imaging studies. Recent studies of alternative energy substrates—glucose and glutamine (Fig. 1)—for aggressive breast cancers provide an example of a research area that is translating to humans and is uniquely enabled by TB PET.

## SUMMARY

The introduction of TB PET systems at several sites throughout the world has been remarkably impactful, with both research and clinical successes. The benefits of TB PET with these first-generation systems became clear after the first human studies (*1* *,* *2*) and have become only more apparent with greater use. Ideally, we will find an optimal AFOV that provides maximum benefit for each application, although system cost and diagnostic yield may temper the widespread adoption of TB PET scanners that truly encompass the entire body. Yet, when appropriately matched to a clinical or research indication, these powerful scanners have the potential to transform patient care and PET research beyond the scope of current investigations to change both science and practice.

## References

[1] Badawi RD Shi H Hu P. First human imaging studies with the EXPLORER total-body PET scanner. J Nucl Med. 2019;60:299–303. 30733314 10.2967/jnumed.119.226498 PMC6424228

[2] Pantel AR Viswanath V Daube-Witherspoon ME. PennPET Explorer: human imaging on a whole-body imager. J Nucl Med. 2020;61:144–151. 31562224 10.2967/jnumed.119.231845 PMC6954463

[3] Alberts I Hünermund JN Prenosil G. Clinical performance of long axial field of view PET/CT: a head-to-head intra-individual comparison of the Biograph Vision Quadra with the Biograph Vision PET/CT. Eur J Nucl Med Mol Imaging. 2021;48:2395–2404. 33797596 10.1007/s00259-021-05282-7 PMC8241747

[4] Surti S Pantel AR Karp JS. Total body PET: why, how, what for? IEEE Trans Radiat Plasma Med Sci. 2020;4:283–292. 33134653 10.1109/trpms.2020.2985403 PMC7595297

[5] Viswanath V Pantel AR Daube-Witherspoon ME. Quantifying bias and precision of kinetic parameter estimation on the PennPET Explorer, a long axial field-of-view scanner. IEEE Trans Radiat Plasma Med Sci. 2020;4:735–749. 33225120 10.1109/trpms.2020.3021315 PMC7673289

[6] Zein SA Karakatsanis NA Issa M Haj-Ali AA Nehmeh SA. Physical performance of a long axial field-of-view PET scanner prototype with sparse rings configuration: a Monte Carlo simulation study. Med Phys. 2020;47:1949–1957. 31985827 10.1002/mp.14046

[7] Daube-Witherspoon ME Viswanath V Werner ME Karp JS. Performance characteristics of long axial field-of-view PET scanners with axial gaps. IEEE Trans Radiat Plasma Med Sci. 2021;5:322–330. 34179595 10.1109/trpms.2020.3027257 PMC8224406

[8] Moskal P Kowalski P Shopa RY. Simulating NEMA characteristics of the modular total-body J-PET scanner: an economic total-body PET from plastic scintillators. Phys Med Biol. 2021;66:175015. 10.1088/1361-6560/ac16bd 34289460

[9] Stockhoff M Decuyper M Van Holen R Vandenberghe S. High-resolution monolithic LYSO detector with 6-layer depth-of-interaction for clinical PET. Phys Med Biol. 2021;66:155014. 10.1088/1361-6560/ac1459 34261049

[10] Zhang X Xie Z Berg E. Total-body dynamic reconstruction and parametric imaging on the uEXPLORER. J Nucl Med. 2020;61:285–291. 31302637 10.2967/jnumed.119.230565 PMC8801950

[11] Wang G Nardo L Parikh M. Total-body PET multiparametric imaging of cancer using a voxel-wise strategy of compartmental modeling. J Nucl Med. November 18, 2021 [Epub ahead of print]. 10.2967/jnumed.121.262668 PMC9364337 34795014

[12] Chitalia R Viswanath V Pantel AR. Functional 4-D clustering for characterizing intratumor heterogeneity in dynamic imaging: evaluation in FDG PET as a prognostic biomarker for breast cancer. Eur J Nucl Med Mol Imaging. 2021;48:3990–4001. 33677641 10.1007/s00259-021-05265-8 PMC8421450

[13] Reader AJ Verhaeghe J. 4D image reconstruction for emission tomography. Phys Med Biol. 2014;59:R371–R418. 25361380 10.1088/0031-9155/59/22/R371

[14] Suchacki KJ Alcaide-Corral CJ Nimale S. A systems-level analysis of total-body PET data reveals complex skeletal metabolism networks in vivo. Front Med (Lausanne). 2021;8:740615. 34616758 10.3389/fmed.2021.740615 PMC8488174

